# The Organon Society of Boston

**Published:** 1888-05

**Authors:** S. A. Kimball

**Affiliations:** Secretary


### THE PROCEEDINGS OF THE ORGANON SOCIETY OF BOSTON.

MARCH 9TH, 1888.

Dr. Wesselhœft being absent Dr. Winn continued the reading from Stratton's edition of the unintentional errors made by allopathic physicians.

Dr. Bell—Cures are certainly made by allopathic physicians. In such cases the large doses which they give act homœopathically.

It is interesting to note that the prominent symptoms of many of our important drugs were observed by prominent physicians long before Hahnemann's time. In these cases which Dr. Winn is reading you will notice how he pits one man against the other. One has seen a remedy *cause* certain symptoms and has noted them; the other has seen this remedy *cure* these same symptoms, but evidently without knowing it could cause them.

Dr. Winn—An allopathic physician of Watertown was called to a case of dysentery in a child; he treated it with drop doses of Ipecac., and the patient speedily recovered.

Dr. Bell—In regard to what is said about drinking some heating fluid after exposure in the sun, I once had a personal experience of that sort. Nine years ago Dr. Wesselhœft and I were walking in the Alps. One day we started to walk twelve miles in the burning sun. When we reached the hotel at the end of the journey I was completely exhausted, and very much heated. So I ordered a glass of hot water, into which I put about a tablespoonful of wine, and within half an hour after taking this I was much relieved.

Dr. Bell—In regard to heat for burns, I made a simple experiment some time ago. I put mustard paste on one finger until it smarted, then I applied Capsicum tincture, which relieved at once.

The introduction being finished, remarks were made upon its truth at the present day, and how well it would suit as a "review of physic," even now, seventy years after it was written.

Dr. Bell—We have now come to the real object of these meetings, the study of the *Organon*, and before beginning this wonderful work, we should understand what sacrifices Hahnemann made for the truth, and what a lover of the truth he was.

He then read selections from the first volume of the *Chronic*

*Diseases*, and also from Dunham's address to the New York Homœopathic Society. He then read the first section of the *Organon*.

Dr. Bell—I think there are two classes of physicians: those who say their mission is to heal the sick, and those who say their duty is to relieve their patient. Now Hahnemann says that the physician's *only* calling is to restore health to the sick.

Dr. Scales—I recall a case of bilious colic which was not immediately relieved by the remedies given, and the next day the patient wanted Morphine. He had had several attacks of the same kind, and all had been relieved by hypodermics, but he was always a long time in getting about again. I explained to him that it might take a little longer to relieve him homœopathically because he had been treated by Morphine before, but that, if he would be patient, when the relief did come it would be permanent, and there would be no bad after effects. He gradually improved, and was about so much sooner and felt generally so much better than ever before after such an attack, that he was perfectly satisfied he had been treated in the right manner.

Dr. Bell then read extracts from Hahnemann's essay on palliation in his *Medicine of Experience*.

Dr. Bell—In regard to section 3, we should tell patients what we expect to cure in them. Some come to us feeling weak, with lack of vigor, chilly, etc. We should tell them how much stronger they can be made, but should not give them the impression that they can be made as robust as anybody.

I think there are some neurotic patients, especially those who have been allopathically drugged, whom we cannot expect to completely cure.

In regard to their symptoms being imaginary, I do not much believe in patients imagining symptoms. A man may think he has a glass leg; that symptom is real to him, and he does not manufacture it. I do not think that nervous patients as a general thing tell us symptoms unless they have them to some extent at least.

Dr. Kennedy—Does not Hahnemann, in speaking of the "recognition of disease," mean to imply that disease is curable?

Dr. Scales—I think he does, excepting old age and the last sickness.

Dr. Bell—In regard to the giving of Morphine or any other palliative in order to keep the case, I wish to say that there is a third class of physicians, those who will do anything for money.

No physician will ever gain anything by giving palliatives to keep his case. If he does not palliate he may lose his patient, but he will always keep the respect of his neighbors as a man who lives up to his principles, and patients who leave him for Morphine often return to be *cured*, a thing the Morphine cannot do.

Several cases of renal colic were related where the patients had been perfectly relieved by the indicated remedy.

Dr. Kennedy—In a recent number of the *Gazette*, in an article on renal colic, the author states that the amount of pain depends upon the nervous condition of the patient, and not on the size of the stone.

A small, smooth stone will often cause much more pain than a large, rough one. Now, the homœopathic remedy relieves the nervous condition of the patient and in that way relieves the pain, and is not this the way in which patients are relieved by the remedy?

Dr. Davis—I had a case once of a man who had had several attacks of severe renal colic. He put himself under my treatment for his general health. While under my care he had one painful attack of renal colic which was relieved by the indicated remedy. Some months after he had a painless attack, in which he passed a stone without pain.

Dr. Bell—This case of Dr. Davis' shows that patients can pass stones without pain. I think the pain depends upon the nervous condition of the patient, which can be relieved.

Dr. Calderwood—Morphine does not always relieve the pain in these cases of renal colic. I remember a case in my younger days when I gave Morphine at such times. The friends of the patient told him he should have an allopath called in who would give him Morphine and relieve him. He was getting Morphine all the time, as much as I dared to give him, but still he was not relieved.

Dr. Bell—I remember a case with all the symptoms of renal colic which came on after the application of a Belladonna plaster to the back. No stone was ever passed, and probably the plaster brought on the attack.

Adjourned to March 23d.

---

### Regular Meeting, March 23d, 1888.

Dr. Bell began at the first section of the *Organon*.

Dr. Wesselhœft—I remember, when a student at the Harvard Medical School, that Dr. Clarke came in one day and said,

"Johnson, what is the first duty of the physician?" "To heal his patient," said Johnson. "No, sir!" said Clarke. Next!" "To make his diagnosis," said the next fellow. "Right," said Clarke; "the first duty is to make your diagnosis; without that you can do nothing."

It reminds me of what I saw in Vienna some time since. One of the surgeons was terribly exercised because "poor Schmitt," a patient, had been running about three weeks without a diagnosis. A terrible state of affairs!

Dr. Bell—I would like to ask Dr. Wesselhœft what he understands by the first two paragraphs?

Dr. Wesselhœft—I think it is very plainly expressed that the only duty of the physician is to heal the sick, and when he cannot cure to palliate homœopathically, always and constantly. The opposite to this is that the highest and only calling of the physician is to relieve pain.

Dr. Thompkins—I think there is a class between: those who will cure if they can, but will palliate to keep their patients.

Dr. Wesselhœft—No physician has ever lost in self-respect or in the respect of the community by honestly saying, "I cannot do those things that I know to be wrong. I can relieve that pain, but my duty is to relieve the patient."

Pain is nothing but a symptom. There may be conditions when a physician ceases to be a healer; for instance, if a man falls to the ground from a four or five story building and is all smashed to pieces; you see that he is dying, that nothing can be done toward healing him. I think under such circumstances that I would clap an ether sponge over his mouth.

Dr. Hastings—I think it is harder to take a family who have been used to mongrel homœopathic treatment than one which has been wholly allopathic.

Dr. Bell—I had a family come to me some years since which had been accustomed to very poor homœopathic treatment. By gradually correcting a thing here and there, and by giving my reasons for not doing certain things to which they had been accustomed, I soon brought them to see that there was a vast difference between true and false Homœopathy, and now they are perfect homœopathists.

Dr. Wesselhœft—I can tell the young doctor never to be alarmed if patients leave them because he is honest, but if he keeps them by being dishonest it will always react on him sometime. In the eyes of the allopaths, who are the respected men? Certainly not those who use means contrary to every truth of Homœopathy. Very few logical men of the old school look for that Utopia in medicine, the union of the two schools that we hear so much about. It can never take place.

The principles of Homœopathy must be upheld, and people must be told that mongrel Homœopathy is not Homœopathy.

Dr. Bell—I tell my patients that I may fail, but that it is my fault, not that of Homœopathy.

Most of the members present had lost patients by not using palliatives, but thought that they had lost nothing by it. Many of the patients had returned, feeling they could trust a man who had principles and stood by them.

The best palliative was considered to be the single remedy homœopathically applied, even in incurable cases, and this was substantiated by numerous cases.

Adjourned to April 5th.

S. A. KIMBALL, *Secretary.*

